# TGF-β as A Master Regulator of Aging-Associated Tissue Fibrosis

**DOI:** 10.14336/AD.2023.0222

**Published:** 2023-10-01

**Authors:** Li-Li Ren, Hua Miao, Yan-Ni Wang, Fei Liu, Ping Li, Ying-Yong Zhao

**Affiliations:** ^1^School of Pharmacy, Zhejiang Chinese Medical University, Hangzhou, Zhejiang, China.; ^2^Department of Urology, Cancer Hospital, Chinese Academy of Medical Sciences and Peking Union Medical College, Beijing, China.; ^3^Beijing Key Lab for Immune-Mediated Inflammatory Diseases, Institute of Clinical Medical Science, Department of Nephrology, China-Japan Friendship Hospital, Beijing, China.

**Keywords:** aging, organ fibrosis, TGF-β, inflammation, cell senescence, natural products

## Abstract

Fibrosis is the abnormal accumulation of extracellular matrix proteins such as collagen and fibronectin. Aging, injury, infections, and inflammation can cause different types of tissue fibrosis. Numerous clinical investigations have shown a correlation between the degree of liver and pulmonary fibrosis in patients and telomere length and mitochondrial DNA content, both of which are signs of aging. Aging involves the gradual loss of tissue function over time, which results in the loss of homeostasis and, ultimately, an organism's fitness. A major feature of aging is the accumulation of senescent cells. Senescent cells abnormally and continuously accumulate in the late stages of life, contributing to age-related fibrosis and tissue deterioration, among other aging characteristics. Furthermore, aging generates chronic inflammation, which results in fibrosis and decreases organ function. This finding suggests that fibrosis and aging are closely related. The transforming growth factor-beta (TGF-β) superfamily plays a crucial role in the physiological and pathological processes of aging, immune regulation, atherosclerosis, and tissue fibrosis. In this review, the functions of TGF-β in normal organs, aging, and fibrotic tissues is discussed: TGF-β signalling is altered with age and is an indicator of pathology associated with tissue fibrosis. In addition, this review discusses the potential targeting of noncoding.

## 1. Introduction

Fibrosis is the aberrant accumulation of extracellular matrix (ECM), which is frequently caused by the body's response to repetitive or chronic tissue injury [1, 2]. Fibrosis can result in the failure of organ structural integrity, functional impairment and even death. The burden of fibrosis is significant, and twenty-five percent of the world's population is affected [3]; the annualized incidence of major fibrosis-related conditions is approaching five percent [2]. Aging is closely related to the progressive decline in organ function [4]. The accumulation of senescent cells is a major feature of organismal aging [5]. The aberrant and chronic accumulation of senescent cells late in life drives age-related disease, tissue deterioration, and tissue fibrosis [6]. Aging is one of the causes of fibrosis in various organs [7]. Aging gives rise to chronic inflammation, which contributes to fibrosis and decreases organ function [8]. Furthermore, fibrosis, which is a marker of tissue aging, is associated with increased oxidative stress, telomere shortening, and mitochondrial dysfunction in the development of a variety of human chronic diseases [9]. The aging rate is associated with the production of high levels of reactive oxygen species (ROS) [10]. Aging-related fibrosis occurs in various diseases, such as vascular disease, idiopathic pulmonary fibrosis (IPF), cardiac fibrosis, liver fibrosis, and chronic kidney disease.

Transforming growth factor-beta (TGF-β) plays a regulatory role in cell proliferation, senescence, apoptosis, inflammatory response, tissue fibrosis, and aging [11]. TGF-β can modulate aging-related fibrosis by regulating inflammation, DNA damage, oxidative stress, and cellular senescence. Moreover, senescent cells can secrete a profibrotic senescence-associated secretory phenotype (SASP), TGF-β [12]. Thus, TGF-β is a potential target for the treatment of aging-associated fibrosis. This review discusses the changing roles of TGF-β in normal, aging, and fibrotic tissues, along with the possibility of therapeutically targeting TGF-β in tissue fibrosis.

## 2. Fibrosis

Fibrosis can damage any organ and is responsible for up to 45% of all fatalities in the industrialized world [2, 13]. Fibrosis is defined as the excessive accumulation of ECM components such as collagen and fibronectin [13]. Fibrosis is an outcome of a dysregulated tissue repair response following many types of tissue injury [14, 15]. SARS-CoV-2, the virus that causes COVID-19, can also cause fibrotic damage to affected tissues, which can last for months or even years after the virus has been eradicated [16].

### 2.1 Mechanism of fibrosis

**Figure 1.**
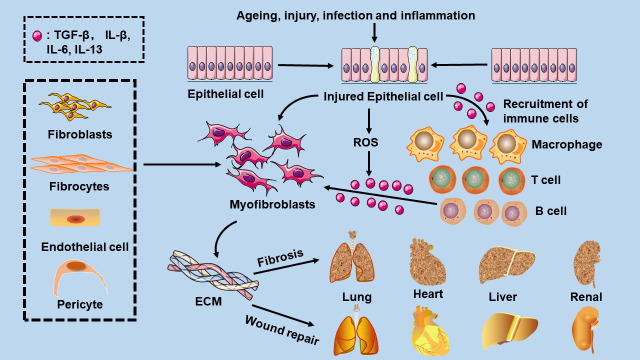
Mechanisms of fibrosis. Aging, injury, infection, and inflammation can promote epithelial cells to myofibroblast transition, regulate ECM formation and remodelling, and ultimately lead to tissue fibrosis or efficient repair. Activated epithelial cells secrete many inflammatory mediators, such as TGF-β1 and interleukin-1β, which recruit immune cells such as mast cells, B cells, T cells, and macrophages. These invasive immune cells release TGF-β, interleukin-1β, interleukin-6, interleukin-13, and other mediators, enhancing profibrotic reactions.

Aging, injury, infections, and inflammation affect whether wound healing results in increased fibrosis or efficient repair [14]. Myofibroblasts can initiate and advance the fibrotic process. In fibrotic illnesses, tissue myofibroblasts come from a variety of sources, such as tissue fibroblasts, and through phenotypic changes in different cell types, such as epithelial cells, endothelial cells, fibrocytes, macrophages, and pericytes [17]. Common features of fibrotic diseases are increased expression of genes encoding ECM proteins, such as fibronectin, cartilage oligomeric matrix proteins, collagen, and tissue inhibitors of metalloproteinases, and decreased activity of ECM-degrading enzymes (Fig. 1).

Macrophages, which are highly heterogeneous cells because of their diverse phenotypes and functions, can have profibrotic effects [18]. Macrophage subpopulations can differentiate into myofibroblasts, which are a source of cytokines and growth factors with fibrotic characteristics and secrete proteases involved in matrix remodelling to regulate fibrosis [19]. Src, a nonreceptor tyrosine kinase, could be activated by TGF-β1. Studies have showed that inhibiting Src activation can inhibit the TGF-β/Smad3-dependent macrophage-myofibroblast transition [20]. Moreover, macrophages eliminate dead cells and encourage the development of reparative fibroblasts to promote the fibrotic process [17].

### 2.2 Fibrosis and inflammation

Tissue damage is associated with some amount of inflammation [13]. Tissue damage and inflammation are important triggers of regeneration and fibrosis. Clinically, inflammaging is characterized by increased blood levels of several inflammatory biomarkers, including interleukin-6 (IL-6), IL-18, and tumour necrosis factor-α [21]. Tissue damage not only induces general inflammation but also determines the type of inflammation by recruiting and activating a variety of different innate and adaptive immune cells [22]. Activated cells secrete proinflammatory cytokines, such as TGF-β1 and IL-1β, which recruit immune cells such as mast cells, B cells, T cells, and macrophages. These invasive immune cells release TGF-β, IL-1β, IL-6, IL-13, and other mediators, enhancing profibrotic and inflammatory reactions [22]. Fibrosis is the ultimate pathological consequence of inflammatory diseases. Multiple inflammatory signals promote the epithelial-mesenchymal transition (EMT) (Fig. 1).

### 2.3 Fibrosis and aging

Furthermore, fibrosis is a sign of aging in tissues, and numerous studies have linked fibrosis to increased oxidative stress in the development of some chronic human diseases [9]. Many clinical studies indicate that telomeres and mitochondrial DNA levels, which are signs of aging, are linked to the degree of liver and pulmonary fibrosis [23, 24]. Tissue fibrosis is mostly seen in elderly individuals. The prevalence of fibrosis in young people is low. Aging is a cause of fibrosis. In aging-related fibrosis, there is long-term abnormal accumulation of senescent cells and a long-term increase in low-grade inflammation [12, 25]. The abnormal accumulation of senescent cells can produce a profibrotic SASP [12]. Moreover, chronically increased low-grade inflammation leads to impaired tissue regeneration and repair [25]. In non-aging-related tissue fibrosis, senescent cells secrete antifibrotic matrix metallopeptidases (MMPs) to degrade ECM components and inhibit fibrosis [26].

## 3. Aging

Aging is the primary risk factor for cancer, cardiovascular disease, diabetes, neurodegenerative disorders, and IPF [27]. The leading causes of damage in aging include telomere damage, impaired nutrient sensing, epigenetic dysregulation, DNA damage, and mitochondrial dysfunction [28]. Furthermore, the aging rate has been associated with the production of high levels of ROS and/or reactive nitrosative species [29]. Aging involves the progressive decline in tissue function over time, which causes the loss of homeostasis and, in turn, the loss of fitness in the organism [28]. In mammals, aging occurs heterogeneously across multiple organ systems, causing progressive deterioration that ultimately results in tissue dysfunction. Aged individuals often exhibit a reduction in the glomerular filtration rate and cortical volume that can result in glomerulosclerosis and nephron atrophy [28].

### 3.1 Cellular senescence

**Figure 2.**
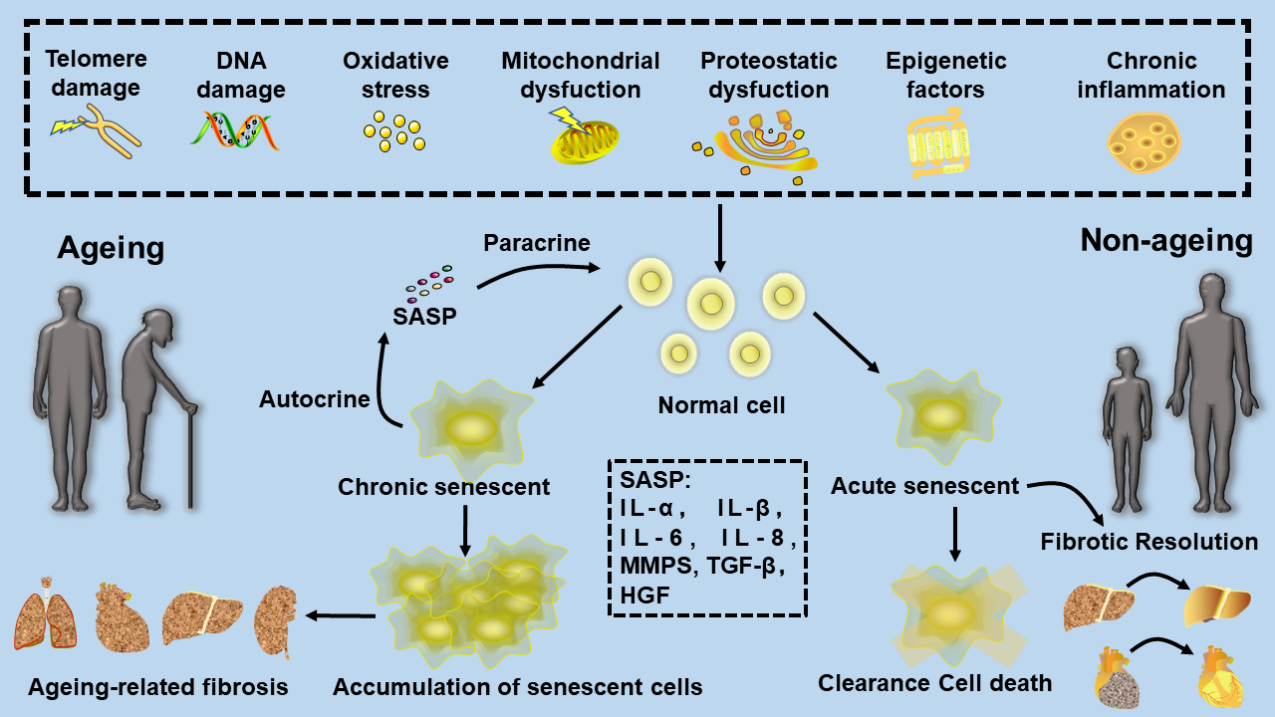
The role of cellular senescence in fibrosis. Cellular senescence is caused by telomere damage, epigenetic dysregulation, DNA damage, oxidative stress, mitochondrial dysfunction, proteostatic dysfunction, and mitochondrial dysfunction. Acute cellular senescence produced early in life provides a benefit during tissue fibrosis, but the aberrant and chronic accumulation of senescent cells late in life drives various features of aging, including age-related fibrosis.

A major feature of aging is the accumulation of senescent cells. Increasing evidence suggests that cellular senescence plays a key role in age-related dysfunction and diseases [5]. Cellular senescence is triggered by a variety of factors, including DNA damage, telomere failure, oncogene activation, and oxidative stress. The senescence program is implicated in various biological processes, including embryogenesis, tissue regeneration and repair, and tumorigenesis [30]. Cellular senescence is characterized by perpetual cell cycle arrest and other phenotypic alterations that include an inflammatory mix of cytokines, growth factors, and MMP, which form the so-called SASP that alters cellular function [31]. This paracrine signalling leads to negative effects on tissue remodelling and chronic inflammation. Cellular senescence early in life provides a benefit during development and tissue regeneration and inhibits neoplastic transformation, but the aberrant and chronic accumulation of senescent cells late in life drives various features of aging, including age-related disease and tissue deterioration [32] (Fig. 2).

#### 3.1.1 Senescence: The molecular players

##### 3.1.1.1 Senescence-associated beta-galactosidase (SA-β-gal)

The accumulation of SA-β-gal is the most widespread biomarker of senescent cells in cultured cells and tissue samples. Senescent cells express SA-β-gal and show high enzyme activity at pH 6.0. The SA-β-gal staining kit uses X-Gal as a substrate and catalyses SA-β-gal to produce a dark blue product. Therefore, a blue colour in cells or tissues expressing SA-β-gal can be easily observed under an optical microscope. The abnormally enlarged and flattened morphology of cells is a characteristic of cellular senescence. As a cell ages, its absorption of substances is reduced, and it is unable to meet its requirements; therefore, the cell becomes larger, and its ability to absorb substances is enhanced, similar to the enlargement of the thyroid gland during iodine deficiency. This change is called compensatory hyperplasia. Research has shown that an increase in cell size may be due to senescence-associated growth arrest. In addition, SA-β-gal-positive senescent cells are increased in size compared with SA-β-gal-negative cells, as identified on a single-cell level in vivo [33].

##### 3.1.1.2 p21 and p16

Senescent cells exhibit permanent cell cycle arrest. The expression of p21 or p16 increases with cellular senescence. The p21 and p16, which are cyclin-dependent kinase inhibitors, are part of the tumour suppressor pathways governed by p53 and are increased in senescent cells. P21 and p16 expression levels are sufficient to establish and maintain senescence-associated growth arrest. Thus, these factors are used to identify senescent cells in tissues and cultured cells [34].

##### 3.1.1.3. IL-6 and IL-8

The transcript and protein levels of SASP components, which are mainly the proinflammatory cytokines IL-6 and IL-8, may be used to evaluate general tissue or cell culture senescence [35].

#### 3.1.2 Inhibitory effect of senescence on fibrosis

Recent findings indicate that the induction of senescence in myofibroblasts can speed up wound healing and limit or reduce myocardial fibrosis, cirrhosis, and IPF [6]. The matricellular protein cysteine-rich angiogenic inducer 61 (CCN1) activates NADPH oxidase 1 (NOX1) through Ras-related C3 botulinum toxin substrate 1 to induce ROS production. These CCN1-induced senescent fibroblasts secrete antifibrotic MMPs to degrade ECM components and ameliorate fibrosis [36].

Senescence significantly improves fibrosis in infarcted hearts (Figure 2). Senescence improves fibrotic problems and cardiac function through the induction of infarcted cardiac senescence by CCN1 [37]. Therefore, as in the case of liver fibrosis, treatments that induce senescence may also be attractive for myocardial infarctions [38]. The administration of CCl_4_ to mice resulted in liver damage, fibrotic scarring, and senescent hepatic stellate cells (HSCs) at the periphery of the scar. These senescent HSCs facilitated fibrotic resolution by decreasing the production of ECM components, as well as increasing the expression of antifibrotic SASP factors such as MMPs [26].

#### 3.1.3 Promotional effect of senescence on fibrosis

Fibroblasts and tissues isolated from IPF patients exhibit increased levels of SA-β-Gal labelling and p21, indicating a relationship between IPF and senescence. In a bleomycin-induced IPF mouse model, the levels of SA-β-Gal were elevated. Bleomycin triggers senescence in fibroblasts and epithelial cells in a mouse model of IPF. Senescent lung fibroblasts may express a profibrotic SASP because they can stimulate myofibroblast differentiation in a paracrine manner (Figure 2). Increases in NOX4 and decreased antioxidant response NFE2-related factor 2 (Nrf2) expression mediate pulmonary senescence. Up to one-third of cases of IPF are thought to be genetically predisposed. Approximately 25% of IPF patients have mutations in telomerase reverse transcriptase and telomerase RNA component genes. These mutations are linked to short telomeres, which could accelerate IPF and cause lung cell senescence [12].

### 3.2 Inflammation and aging

Inflammation is one of the most described aging-related phenotypes [25]. With increasing age, individuals develop a persistent, low-grade, subclinical proinflammatory status [39]. ROS produced by dysfunctional mitochondria can also trigger an inflammatory response by activating nuclear factor-kappa B (NF-κB) [40]. Cellular senescence activates innate and adaptive immune responses. This chronic low-grade inflammation that increases with age is also known as “inflammaging” [41]. Accumulating evidence has shown that inflammaging is a risk factor for impaired tissue regeneration and repair, which is associated with many aging-related diseases [42].

## 4. TGF-β

TGF-β is a versatile cytokine that can be produced by different cell types, such as platelets, monocytes, macrophages, T cells, and fibroblasts [43]. The TGF-β superfamily contains more than 30 members, such as TGF-β, bone morphogenic proteins, activins, inhibins, nodal, myostatin, growth and differentiation factors, and anti-Mullerian hormone [44]. TGF-β signalling regulates a wide range of cellular functions, as well as fundamental biological processes such as embryonic development, immune response, wound healing, and angiogenesis [45]. The TGF-β superfamily has been shown to play an important role in a wide range of physiological and pathological processes, including aging, immune regulation, atherosclerosis, and tissue fibrosis [46, 47].

### 4.1 TGF-β in fibrosis

#### 4.1.1 The TGF-β signalling pathway in fibrosis

**Figure 3.**
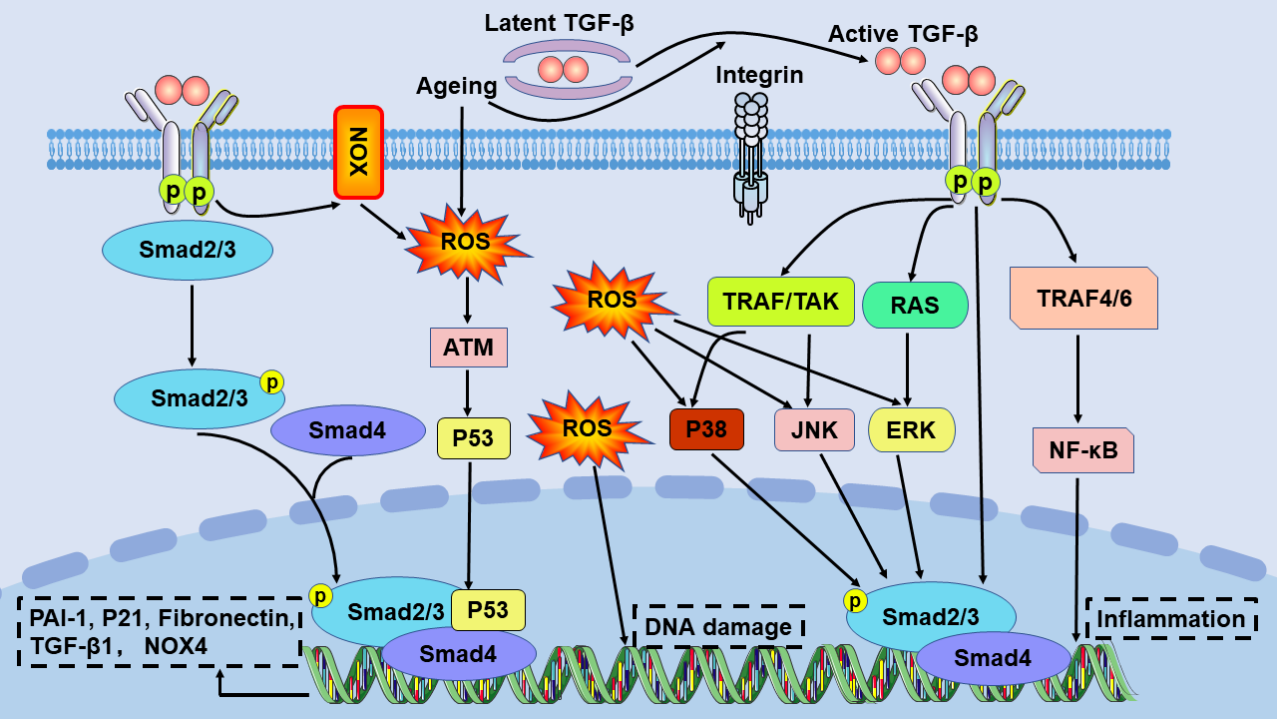
The TGF-β signalling pathway in aging and fibrosis. TGF-β is an inactive complex formed by the combination of TGF-β with a latency-associated peptide. Latent activation of TGF-β in fibrotic illnesses may involve ROS, aging, and integrins. NOX is one of the key enzymes linking ROS and TGF-β activity. Different NOX subtypes contribute to the production of intracellular ROS. ROS overproduction can lead to DNA damage. ROS interacts with classical and nonclassical TGF-β signalling pathways to regulate inflammation and the expression of the PAI-1, P21, fibronectin, TGF-β, and NOX4 genes. ATM, ataxia telangiectasia mutated protein; JNK, c-Jun terminal kinase; NF-κB, nuclear factor-kappa B; NOX, NADPH oxidase; PAI-1, plasminogen activator inhibitor-1; ROS, reactive oxygen species; TAK1, TGF-β-activated kinase 1; TRAF, TGF-β receptor-associated factor.

TGF-β binds to latency-associated peptides when TGF-β is not activated. It has been shown that the binding of latent TGF-β-binding protein to latency-associated peptide facilitates the storage of complexes in the ECM [48]. TGF-β may be activated by ROS, platelet response protein 1, proteases, aging, and integrins. TGF-β is an important regulator of tissue fibrosis and tissue scarring by activating the small downstream mother against the decapentaplegic (Smad) signalling pathway. TGF-β binds to a TGF-β receptor II (TβRII) dimer and two TGF-β receptor I (TβRI) molecules and then phosphorylates receptor-activated Smad, Smad2, and Smad3. Receptor-activated Smad oligomerizes with Smad4 to form a trimeric protein complex. These complexes are transferred to the nucleus, where they bind to other transcription factors to induce or inhibit the expression of fibrosis-related genes [49, 50] (Figure 3). Smad7, which is a member of the inhibitory Smad family, is part of a conserved negative feedback loop in most animals [48]. bone morphogenic protein-7 inhibits TGF-β1-induced tubular EMT in mice by antagonizing TGF-β1-induced downregulation of E-cadherin and TGF-β1-induced upregulation of α-smooth muscle actin (α-SMA) expression [51].

The TGF-β1/Smad signalling pathway is connected to many different pathways, such as MAPK, phosphoinositide 3-kinase/protein kinase B/mammalian target of rapamycin, p53, connective tissue growth factor (CTGF) and Wnt/β-catenin signalling [52].

#### 4.1.2 TGF-β regulates fibrosis by mediating inflammation

At the wound site, inflammatory cells simultaneously perform many tasks by promoting wound debridement and generating chemokines, metabolites, and growth factors. The wound may become chronic or gradually fibrotic if this carefully managed reaction becomes dysregulated [22]. TGF-β1 has long been identified as a critical mediator of tissue repair and fibrosis after type 1 and type 17-driven inflammatory responses, which are characterized by the production of the proinflammatory cytokines interferon-γ and IL-17A, respectively. Moreover, several studies have shown that the IL-17A/IL-1β/TGF-β1 axis is important for the development of fibrosis [53].

### 4.2 TGF-β signalling in aging

The exposure of cultured epithelial cells, endothelial cells, haematopoietic cells, and keratinocytes to TGF-β results in cell cycle arrest [54]. Downstream target regulation of TGF-β signalling is involved in multiple aspects of aging, including cell proliferation, cell cycle regulation, the generation of ROS, DNA damage repair, telomere regulation, oncogene activation, and autophagy [55].

#### 4.2.1 TGF-β in DNA damage

Canonical TGF-β signalling promotes senescence through the loss of H4K20me3 via miRNA-29. The TGF-β signalling pathway causes DNA damage in two ways: an acute increase in miRNA-29a and miRNA-29c to inhibit the novel target suf4-20h and reducing H4K20me3 in a Smad-dependent manner. In vivo, cardiac aging is caused by H4K20me3 deficiency, which is driven by the senescent TGF-β/miRNA-29 pathway [56].

#### 4.2.2 TGF-β and ROS

Mechanistically, oxidative stress can activate TGF-β signalling. Increased ROS levels cause DNA damage and senescence. It is well known that ROS and TGF-β are interconnected [10]. ROS formation is enhanced in response to TGF-β1, possibly due to the inhibition of the expression of several antioxidant enzymes. On the other hand, elevated levels of ROS lead to proteolytic activation and increased production of TGF-β1 in different organs. It was recognized that different NOXs are critical in linking ROS and TGF-β activity. Different NOX subtypes contribute to the production of intracellular ROS [57]. A study showed that NOX1- or NOX4-deficient mice were protected against liver inflammation and fibrosis. Furthermore, the depletion of the regulatory p47phox subunit of NOX, which mediates ROS production, resulted in the amelioration of renal fibrosis following injury [58]. The aging process was shown to strongly enhance the activities of ERK, C-Jun terminal kinase, and p38 MAPK, which paralleled increases in ROS production [59] (Fig. 3).

#### 4.2.3 TGF-β and cellular senescence

TGF-β signalling can regulate cellular senescence by inducing the cyclin-dependent kinase inhibitors p15, p21, and p27 and by suppressing several proliferation factors, including c-Myc [54] (Fig. 3).

The p53 tumour suppressor is the most researched mediator of cellular senescence. P53 can interact directly with Smad2 and Smad3, which activate genes containing p53-binding sites and TGF-β responsive regions, such as the gene encoding p21 [54].

The telomerase (hTERT) gene can prevent types of senescence related to telomere shortening. TGF-β signalling leads to transcriptional downregulation of hTERT expression in colon and breast cancer cells. The oncogene c-Myc governs hTERT transcription in many human malignancies. TGF-β indirectly modulates hTERT expression by suppressing c-Myc expression. In these contexts, Smad3 can interact directly with the hTERT promoter, resulting in the direct suppression of hTERT transcription.

An increase in TGF-β leads to cell cycle arrest, telomere shortening, and DNA damage in senescent cells. In addition, an increase in TGF-β inhibits the proliferation of immunocompetent cells; inhibits the differentiation of lymphocytes; promotes the growth of fibroblasts, osteoblasts, and Schwann cells; inhibits the growth of epithelial cells, osteoclasts, and endothelial cells; inhibits the formation of adipose tissue, cardiac tissue and skeletal muscle; inhibits the adhesion of lymphocytes to endothelial cells; and promotes the release of histamine from basophils.

### 4.3 TGF-β and inflammation

Integrin αvβ6, which is expressed by epithelial cells, and αvβ8, which is expressed by dendritic cells, play key roles in the activation of TGF-β. Once activated, TGF-β plays an important role in controlling anti-inflammatory and proinflammatory T-cell responses, based upon the amount of TGF-β that is released by the cells, as well as the intracellular and local extracellular factors that are present at the time of TGF-β exposure [60].

#### 4.3.1 Anti-inflammatory effects of TGF-β

TGF-β exerts its anti-inflammatory effect by inhibiting Th1 cells and Th2 cells and promoting the differentiation of Treg cells [60]. TGF-β effectively inhibits Th1 cell differentiation by inhibiting the production of T-BET and downregulating the expression of IL-12Rβ2 on T cells, thereby reducing their reactivity to IL-12, and inhibits the production of IFN-γ by natural killer cells to indirectly inhibit Th1 cell differentiation [61]. The transcription factor GATA3 is a key transcription factor in Th2 cell differentiation. TGF-β downregulates the expression of the transcription factor GATA3 and effectively downregulates Th2 cell differentiation by inducing the transcription factor Sox4 to bind to the IL-5 promoter and block GATA3-mediated gene expression [60]. TGF-β promotes the transformation of naive CD4+ T cells into induced Tregs by upregulating Foxp3 expression. Smad2 and Smad3 promote Foxp3 induction through different mechanisms.

Smad7 is an inhibitory factor that blocks Smad2/3 activation by degrading TβRI and Smad2/3 and inhibits the NF-κB-driven inflammatory response by inducing IκBα, an inhibitor of NF-κB [62].

#### 4.3.2 Proinflammatory effects of TGF-β

TGF-β induces the production of IL-17 and the expression of the transcription factor RORγt, which are essential for the generation of Th17 cells (CD4+ T cells). Th17 cells are essential for the development of inflammation and the immune response to specific extracellular bacteria and fungi. TGF-β requires proinflammatory cytokines such as IL-6, IL-1, and IL-21 to induce Th17 cells [63].

Th9 cells are a subpopulation of proinflammatory CD4+ T cells that release IL-9 and can drive inflammation in a variety of diseases. TGF-β can induce Th9 cells. TGF-β induces the essential transcription factor PU.1, which is necessary for the induction of Th9 cells in the presence of IL-4 [64].

### 4.4 Aging-associated Sirtuin1 (SIRT1)/5'-AMP-activated protein kinase (AMPK)/autophagy regulates fibrosis by modulating TGF-β

SIRT1, which is a NAD+-dependent histone deacetylase that has a variety of molecular functions, has become a key protein in the modulation of aging and metabolism. Nuclear SIRT1 is identified by the autophagy protein LC3 as an autophagy substrate during aging and is subjected to cytoplasmic autophagosome lysosome degradation [65]. Research has demonstrated that SIRT1 can prevent TGF-β-induced EMT in human endothelial cells by de-acetylating Smad4 [66]. Additionally, SIRT1 overexpression could attenuate TGF-β1-induced renal fibrosis in vitro [67].

In many animals, including humans, nutritional intervention can alleviate age-related illnesses and slow the aging process [68]. AMPK is a crucial mediator of nutritionally sensitive pathways. In mammals, AMPK activation decreases with age. AMPK signalling can regulate the symptoms of aging, such as autophagy, SIRT1, endoplasmic stress, oxidative stress, DNA damage repair, mitochondrial dysfunction, and inflammation [69]. AMPK activation can lower the activity of the TGF-β1 promoter and impair TGF-β-induced Smad phosphorylation to reduce TGF-β1-induced collagen Ι production [70].

Autophagy is a process by which cytoplasmic components, including macromolecules (such as proteins, glycogens, lipids, and nucleotides) and organelles (such as mitochondria, peroxisomes, and the endoplasmic reticulum) are degraded by the lysosome. Autophagy plays a crucial role in maintaining cellular homeostasis in response to intracellular stress. However, the effectiveness of autophagy declines with age, and excess nutrient intake can impair the autophagic process [71]. TGF-β increases the expression of the autophagy-related genes Atg5, Atg7, and Beclin 1 to activate autophagy through SMAD3-dependent downregulation of the H4K16 histone acetyltransferase MYST1 in fibrotic illnesses, augmenting its profibrotic effects [72]. Additionally, TGF-β1 enhances apoptosis in tubular cells and triggers autophagy by generating ROS [73].

## 5. The role of TGF-β signalling in aging-related tissue fibrosis

Aging leads to the loss of function in tissues and/or cells in all organisms [27]. Senescent cells secrete the profibrotic SASP [12]. In addition, numerous clinical studies have shown that telomere and mitochondrial DNA levels are biomarkers of aging and correlate with the degree of tissue fibrosis [23]. This finding suggests that aging and organ fibrosis are closely related. Chronic hyperactivation of TGF-β signalling causes aging-related fibrotic diseases [74]. Aging results in the activation of TGF-β signalling in the brain, corneal epithelium, heart, lung, kidney, and liver [75]. This in-depth review discusses the underlying cellular and molecular mechanisms of the TGF-β signalling pathway in aging-associated fibrosis.

### 5.1 Aging-related vascular fibrosis

Aging is the main risk factor for vascular fibrosis and cardiovascular fibrosis, which is the leading cause of death worldwide [76]. Aging can cause alterations in endothelial morphology and dysfunction that result in arterial stiffness, atherosclerosis, hypertension, stroke, and coronary artery disease [77]. Fibrosis and remodelling of the ECM are the primary causes of vascular stiffness [76]. Vascular wall thickening, reduced vasodilation and arteriosclerosis occur as a result of aging. Cardiovascular function decreases with age as the arterial tree thickens and hardens [78]. The sclerosis of large vessels causes haemodynamic harm to the tissues in the surrounding area, such as the heart, kidneys, and brain [58].

Multiple target organs, including the liver, kidney, and heart, developed fibrosis lesions after an intravascular injection of TGF-β in rats [51]. Aging causes an increase in TGF-β1 activation and receptor-mediated signalling in the aortic wall. Importantly, these conditions are characterized by high levels of angiotensin II, mechanical stress, and ROS, which are all known to induce TGF-β activation and the subsequent development of vascular fibrosis [76]. Plasminogen activator inhibitor-1 (PAI-1) is necessary and sufficient for the induction of p53-dependent replicative senescence. PAI-1, a major TGF-β1/p53 target gene and negative regulator of the plasmin-based pericellular proteolytic cascade, is elevated in arterial plaques, vessel fibrosis, arteriosclerosis, and thrombosis and correlates with increased tissue TGF-β1 levels [79]. TGF-β-driven vascular cell regulation may play a role in the aetiology of fibrosis, according to the literature. The effect of TGF-β on endothelial cells and parietal cells can result in the development of fibrous phenotypes, and the cells express ECM proteins and secrete fibroblast activation mediators. Furthermore, under the influence of TGF-β, endothelial cells and vascular wall cells can differentiate into fibroblasts, increasing the number of cells that produce extracellular matrix in fibrotic tissues [80].

### 5.2 Aging-related IPF

IPF is a progressive, lethal fibrotic lung disease that can be caused by fibrous remodelling. IPF remodelling is characterized by extreme difficulty breathing and coughing, as well as a median survival duration of 3.8 years [81]. IPF is considered to be an aging illness because it exhibits overlaps between pathways associated with biological aging [82]. Research shows that fibrotic lung disease is mediated in part by senescent cells [83]. Gene mutations (surfactant proteins B and C, mucin 5B, telomerase) trigger functionally debilitating lung lesions that are fatal within 3-5 years of diagnosis [84]. A study showed an increased abundance of aging biomarkers in IPF lungs, p16 expression was increased with disease severity, and the deletion of senescent cells restored lung health in aged mice [83]. Alveolar senescent epithelial cells, which are characterized by telomere shortening, oxidative damage, dysregulated protein inhibition and endoplasmic reticulum stress, and mitochondrial dysfunction, can contribute to the central phenotype of pulmonary fibrosis pathogenesis, which results in increased alveolar epithelial cell proliferation and the secretion of profibrotic mediators [85]. Individuals over the age of 75 had an approximately eightfold greater incidence of pulmonary fibrosis than patients under the age of 55, according to previous studies [13].

TGF-β is a central mediator of IPF, according to a growing body of evidence [82]. Extracellular heat shock protein 90α activates TGF-β signalling through the phosphorylation of the Smad complex, whose binding to the p53 and p21 promoters triggers their transcription, mediates fibroblast senescence, and promotes mitochondrial dysfunction in IPF [86]. Studies have shown that TGF-β1/IL-11/MEK/ERK signalling mediates aging-related pulmonary fibrosis in B-cell-specific Moloney murine leukaemia virus insertion region 1-deficient mice [87]. TGF-β1/IL-11/MEK/ERK signalling stimulated cellular senescence, profibrotic SASP, and EMT in alveolar type II epithelial cells. Due to its various roles in cell-based signalling pathways, the TGF-β signalling pathway is crucial in lung development and physiology [88]. TGF-β is the primary mediator of the pathophysiology of fibroblast accumulation and myofibroblast differentiation [2]. Although TGF-β is expressed by a range of cell types in the lung, including epithelial cells, macrophages, and fibroblasts, it is elevated in animal models of IPF, as well as in the lungs of IPF patients [89]. As a result of lung epithelial cell damage and exposure to the alveolar basement membrane, TGF-β1 accumulates, which stimulates fibroblast recruitment and the creation of ECM [90].

### 5.3 Aging-related cardiac fibrosis

Cardiac fibrosis is a pathological ECM remodelling process that causes aberrant matrix composition, as well as decreased cardiac function [91]. Cardiac fibroblasts, which maintain extracellular matrix homeostasis, differentiate into myofibroblasts after damage and promote cardiac fibrosis [92, 93]. Cardiac fibrosis is a common pathophysiologic companion of most cardiac illnesses, and it is linked to systolic and diastolic dysfunction, arrhythmogenesis, and poor prognosis. The pathological symptoms of many cardiac illnesses, such as myocardial infarction, hypertension, myocarditis, hypertrophy, and dilated cardiomyopathy, can result in myocardial fibrosis, which can lead to end-stage cardiac failure [94]. Cardiac failure and accompanying morbidity and mortality are on the rise at an alarming rate [95]. Aging may cause interstitial and perivascular fibrosis in the heart. The gradual accumulation of collagen in the heart with age leads to ventricular stiffness and impaired diastolic function. Arterial compliance is reduced, leading to the direct fibrotic effects associated with aging [96]. Aging is an important predisposing factor for cardiac fibrosis [97]. Aging-related processes such as inflammation, autophagy, and mitochondrial dysfunction are interconnected biological processes that decrease the regenerative capacity of aged cardiac tissue and have been shown to play crucial roles in cardiac fibrosis [96]. The heart is also highly susceptible to cumulative oxidative damage and stress with age due to its high metabolic demands and high mitochondrial and infrequent cardiomyocyte turnover [97].

The prevalence of cardiac fibrosis increases dramatically with age [6]. TGF-β activation mediates interstitial and perivascular fibrosis in aging cardiac tissue [96]. Studies have shown that TGF-β1-p38 signalling promotes age-related cardiac fibrosis. Increased microRNA (miRNA)-1468-3p levels in human cardiac fibroblasts increases SA-β-gal activity, p53, and P16 expression and enhances collagen I and CTGF expression [98]. Upregulated miRNA-1468-3p levels in human cardiac fibroblasts increase SA-β-gal activity, p53, and P16 expression, and enhance collagen I and CTGF expression. In the heart, the TGF-β signalling pathway is critical in the activation of cardiac fibroblasts, resulting in the deposition of cardiac ECM and the remodelling of the heart. TGF-β promotes cardiac fibroblast transformation after cardiac injury [99].

### 5.4 Aging-related liver fibrosis

Liver fibrosis is a global health issue that leads to cirrhosis, liver failure, and hepatocellular cancer [100]. Repair mechanisms are activated after liver injury to replace necrotic and apoptotic liver cells, resulting in wound healing and inflammatory responses that are necessary for liver regeneration. Extracellular matrix proteins, including collagen I, II, and III wave proteins, fibronectin, laminin, elastin, proteoglycan, and valine, can replace parenchymal tissue if the damage is severe enough, resulting in fibrosis. Recent findings suggest a link between cellular senescence and liver fibrosis. Senescence in hepatocytes and cholangiocytes is a powerful driver of liver fibrogenesis [23]. Many clinical studies have shown that telomere and mitochondrial DNA levels are senescent biomarkers and correlate with the degree of liver fibrosis in patients with chronic liver disease [23]. Cellular senescence is a significant feature of aging [97]. Senescence is associated with progressive changes in liver structure and the physiological/ pathological functions of liver cells, including hepatocytes, bile duct cells, HSCs, and hepatic sinusoidal endothelial cells. Aging can accelerate the development of liver fibrosis [44].

TGF-β has been identified as a key player in the SASP [101]. In the liver, TGF-β promotes cellular senescence in acute and chronic liver injury models [102]. The TGF-β signalling pathway, which regulates hepatocyte differentiation and death, is involved in all stages of disease progression, from initial liver injury to inflammation and aging-related fibrosis [103]. Studies have shown evidence of active TGF-β signalling in senescent hepatocytes adjacent to necrotic areas after acute liver injury [104]. TGF-β also promotes hepatocyte EMT, which can directly or indirectly increase the number of myofibroblasts [105].

### 5.5 Aging-related renal fibrosis

Renal fibrosis, characterized by tubulointerstitial fibrosis and glomerulosclerosis, is the final manifestation of chronic kidney disease, which was associated with a various molecular mechanisms such as activations of aryl hydrocarbon receptor [106-110], NF-κB [111, 112] and Wnt/β-catenin [113-115] signalling pathways as well as dysbiosis of gut microbiota [116, 117] and disorder of endogenous metabolite [118-122]. Kidney aging has been identified as a chronic process characterized by impaired renal function and structural changes in the tubular interstitium and glomerulus [123]. Cell senescence is associated with alterations in cell structure and function, including the expression of cytokines and structural and regulatory components of ECM proteins. Senescent cells accumulate in the kidney during natural aging and after injury. Glomerulosclerosis and interstitial fibrosis worsen with age, and the glomerular filtration rate decreases [124]. The expression of the fibrosis marker α-SMA is positively correlated with the expression of the senescence marker SA-β-gal and the senescence-associated cell cycle protein-dependent kinase inhibitors p16 and p21 [125]. Further investigation of the mechanisms associated with renal fibrosis revealed that cellular senescence, chronic inflammation, oxidative stress, abnormal autophagy, epigenetic disorders, disturbances in energy metabolism, and excessive or insufficient apoptosis contribute to renal fibrosis. Tubular interstitial fibrosis is a chronic and progressive process that affects aging renal tissue, regardless of the cause. Renal fibrosis is a global health burden with limited therapeutic options. Patients with renal fibrosis have signs of premature aging, such as osteoporosis, poor wound healing, and inflammation [126]. Renal fibrosis is defined as excessive deposition of the extracellular matrix, which destroys and replaces functional parenchyma, leading to organ failure [127]. Renal fibrosis affects more than 60% of those over the age of 80 [128]. The microanatomical structure of the kidney changes with age due to increased nephrosclerosis (arteriosclerosis, glomerulosclerosis, tubular atrophy with interstitial fibrosis), a reduced number of functional glomeruli, and, to some extent, compensatory hypertrophy of residual nephrons [129].

TGF-β is an important SASP factor that has been extensively studied and can interact with other cytokines or molecules involved in fibroblast activation. TGF-β can be activated by ROS [130]. Research shows that tubular cells undergo senescence and express increased TGF-β1 with advancing age. Incremental load training could improve aging-related renal fibrosis in mice by modulating the TGF-β1/TGF-β-activated kinase 1/MAPK kinase/p38 MAPK signalling pathway, activating autophagy, reducing ECM synthesis, and delaying EMT [131]. TGF-β1 overexpression in the kidney results in collagen I and collagen III deposition and mild mesangial proliferation [50]. TGF-β1 causes excessive ECM production and deposition in the glomeruli and tubular interstitium, as well as tubular and glomerular EMT.

## 6. Targeting TGF-β in aging-related fibrosis

TGF-β plays an important role in the physiological and pathological processes of aging-associated fibrosis. Therefore, targeting TGF-β signalling is effective in the treatment of fibrosis associated with aging. In this review, we discuss various strategies for targeting TGF-β signalling in the treatment of aging-related fibrosis.

### 6.1 Targeting noncoding RNAs

MicroRNAs (miRNAs), which are small regulatory noncoding RNAs approximately 22 nucleotides in length, regulate a wide range of cellular pathways [92]. MiRNAs are crucial components of the downstream signalling cascade of TGF-β [132]. TGF-β signal transduction regulates miRNA expression, and miRNAs regulate TGF-β signal transduction to regulate EMT [77].

MiRNA-181 inhibits the TGF-β and pSmad2/3 pathways. This factor plays a key role in ECM remodelling. A study showed that miRNA-181b expression was significantly reduced in the aortas of aged mice [133]. Pulse wave velocity, blood pressure, and vascular stiffness were significantly increased in miRNA-181b-deficient mice. MiRNA-181b may be a therapeutic target to modulate aging-associated vascular stiffness to treat hypertension [133].

Treatment of senescent cells with embryonic stem cell-derived mmu-miRNA-291a-3p reduces senescence-associated β-galactosidase activity and decreases the mRNA and protein expression of TGF-βRII, p53, and p21. Hsa-miRNA-371a-3p and Hsa-miRNA-520e are human homologues of mmu-miRNA-291a-3p. ESC-derived mmu-miRNA-291a-3p is a novel candidate for cell-free treatment of aging and aging-associated fibrosis [134].

Long noncoding RNAs (lncRNAs) are noncoding RNA transcripts longer than 200 bp. LncRNA-was is the first lncRNA that was shown to be activated by TGF-β and promotes epithelial-mesenchymal transition by regulating the TGF-β and miRNA 200 family [135]. Overexpression of lncRNA-ATB enhances the activation of the TGF-β/Smad2/3 signalling pathway by TGF-β1 and promotes TGF-β1-induced inflammation, apoptosis, and senescence in HK-2 cells [136]. Silencing lncRNA-ATB may be a novel strategy for the treatment of senescence-associated renal fibrosis.

A study demonstrated that LncRNA MALAT1 was decreased with age and oxidative stress. LncRNA MALAT1 silencing dramatically enhanced the expression of TGF-β1 in C2C12 cells [137]. In addition, H_2_ O_2_ treatment of C2C12 cells significantly decreased MALAT1 expression. Therefore, targeting lncRNA MALAT1 can treat age-related fibrosis.

### 6.2 Potential therapeutic targets and treatments

TGF-β interacts with other components in the body, and targeting other components can modulate the TGF-β signalling pathway, thereby treating aging-related fibrosis.

Heat shock protein 90 is a ubiquitously expressed chaperone that is involved in the posttranslational folding and stability of proteins. Heat shock protein 90, which has an extracellular function, has two isotypes: inducible alpha and beta [138]. Extracellular heat shock protein 90α (eHSP90α) phosphorylates the Smad complex, and the binding of the Smad complex to the p53 and p21 promoters initiates transcription. eHSP90α was upregulated in bleomycin-induced mice and correlated with the expression of the cellular senescence biomarkers P53 and P21. An increase in eHSP90α mediated senescence in fibroblasts and promoted mitochondrial dysfunction. In vivo, blocking eHSP90α in aged mice with 1G6-D7, an antibody specific for eHSP90α, attenuated bleomycin-induced senescence-associated pulmonary fibrosis [86].

The klotho gene is an antiaging gene. This gene could improve chronic kidney disease and some cardiovascular diseases. Studies have shown that the formation of calcified nodules in the aortic valves of klotho-deficient mice is associated with increased expression of TGF-β1 and elevated levels of the activated forms of Smad2, Smad3, Smad1/5, p38, and ERK1/2 MAPK [139]. Klotho protein can be used to treat aging-related vascular fibrosis.

PAI-1, a member of the serine protease inhibitor superfamily that has antiprotease activity, is crucially involved in fibrinolysis and wound healing [140]. PAI-1 expression increases with age and plays a key role in pulmonary fibrosis in many diseases associated with senescence, including IPF. Deleting PAI-1 or inhibiting PAI-1 activity in alveolar type II cells almost completely blocked TGF-β1-induced senescence in alveolar type II cells, the secretion of profibrotic mediators and the stimulatory effect of the SASP on macrophages [141]. Targeting PAI-1 may be an effective therapeutic strategy for the treatment of IPF, as well as other senescence-related diseases. The PAI-1 inhibitor TM5275 attenuated TGF-β1-induced pulmonary fibrosis and alveolar type II cell senescence in mice [142].

Caveolin-1 is a major membrane-intrinsic protein that maintains caveolae integrity. Studies suggest that Caveolin-1 is involved in controlling the expression of specific TGF-β1/p53-responsive growth arrest genes. The upregulation of Caveolin-1 could arrest cells at G0/G1 by activating the p53/p21 cell cycle arrest pathway. Furthermore, the restoration of Caveolin-1 in Caveolin-1-deficient cells rescued the TGF-β1-induced PAI-1 gene. Molecular events in senescence-associated PAI-1 expression in response to TGF-β1/src kinase/p53 signalling may provide new targets for the treatment of cardiovascular sclerosis [79].

Ubiquitin-specific protease 9x (USP9X) is one of the largest deubiquitinating enzymes in the USP family [143]. USP9X is closely related to oxidative stress and the TGF-β/Smad pathway. Research shows that late glycation end-products dose- and time-dependently reduced the protein expression of USP9X and deubiquitinase activity in glomerular mesangial cells. USP9X overexpression attenuated late glycation end-product-induced fibronectin, TGF-β 1, and collagen IV in a deubiquitinase activity-dependent manner. Silencing USP9X with siRNAs further promoted the expression of these proteins in late glycation end-product-treated glomerular mesangial cells [144]. USP9X can be used to ameliorate the pathological process of aging-associated diabetic renal fibrosis.

USP11 belongs to the cysteine protease family. Studies have shown that an increase in USP11 in pathological states is associated with the promotion of renal fibrosis. USP11 phosphorylates TβRΙ by deubiquitinating TβRII and reducing TβRII ubiquitination degradation, which then activates the downstream Smad2/3 or nonclassical pathway to promote the activation of downstream senescence signalling pathways and the development of renal fibrosis [145].

Nerve growth factor-inducible gene B (Nur77) is a nuclear receptor that regulates inflammatory diseases. Nur77 ameliorates several biological processes in chronic diseases, including inflammatory responses, cell proliferation, and metabolism. Studies have shown that Nur77 significantly inhibits the TGF-β/Smad signalling pathway and stabilizes the homeostasis of the Smad7 protein in the treatment of senile nephropathy and tubulointerstitial fibrosis [146].

The inhibitor of kappa B kinase beta (Ikkβ) is a transcription factor-activated protein that is involved in a variety of intracellular signalling pathways, including apoptosis caused by cytokines. Studies have shown that in vitro ablation of Ikkβ in fibroblasts leads to the progressive accumulation of ROS and activation of TGF-β and ultimately accelerates cell migration, fibroblast-myofibroblast transformation, and senescence. Ikkβ-deficient cells show ROS accumulation and activation of the stress-sensitive transcription factor AP-1/c-Jun. The activation of AP-1/c-Jun leads to the upregulation of TGF-β [147]. Ikkβ plays a key role in preventing fibroblast-myofibroblast transformation and senescence.

Androgens, which are a group of sex hormones, help initiate puberty and play a role in reproductive health and body development. Androgens can modulate ECM accumulation and oxidative stress. Circulating testo-sterone levels increase with age and are progressively reduced in male rats. Renal fibrosis increases in aged rats, which is characterized by thickening of the glomerular basement membrane and Bowman's basement membrane, increased ECM, decreased MMP-2 and MMP-9 expression, increased TIMP-1 and TIMP-2 expression, increased TGF-β1/Smad signalling, and decreased Nrf2-ARE signalling in renal tissue compared to those in younger rats. Subcutaneous administration of testosterone propionate significantly improved these age-related indicators [148]. Testosterone propionate may ameliorate aging-associated renal fibrosis in aged rats by inhibiting TGF-β1/Smad signalling and activating Nrf2-ARE signalling.

Integrin β3 is one of the main integrin heterodimer receptors on the surface of cardiac myocytes [149]. Integrin β3, which is regulated by the Polycomb protein CBX7, accelerates the onset of senescence in human primary fibroblasts by activating the TGF-β pathway in a cell-autonomous and noncell-autonomous manner. The integrin β3 antagonist cilengitide blocks the SASP without affecting proliferation [150]. Because senescent cells accumulate during aging, which leads to chronic inflammation and organ fibrosis, cilengitide may be a potential therapeutic agent to block aging-associated fibrosis without affecting proliferation.

**Table 1:** Natural products that inhibit TGF-β signalling in aging-related tissue fibrosis.

Compounds	Targets	Outcomes	Ref.
Saponins from *Panax japonicus*	Regulate the TGF-β1/Smad pathway	Improved aging-related renal fibrosis	[151]
Mangiferin	Regulate the TGF-β1/p38/MAPK pathway	Improved D-galactose-induced cardiac fibrosis in aging rats	[152]
Amylase Potato Protein Hydrolysate	Regulate TGF-β expression	Improved high fat diet- and aging-induced cardiac hypertrophy and fibrosis	[153]
Metformin	Regulate TGF-β signalling	Improved aging-related fibrosis (e.g., chronic kidney disease, nonalcoholic steatohepatitis, cardiac failure, or sclerosis)	[155]
Date palm seeds	Inhibit IL-1β and TGF-β expression	Increased immunity: prevention of chronic diseases to improve aging-related fibrosis	[156]
Xiaoyu Xiezhuo Drink	Regulate the TGF-β1/Smad3 pathway	Alleviation of cellular senescence, inflammation, and oxidative damage to improve aging-related renal fibrosis	[157]

### 6.3 Natural products

Studies show that some natural products can treat aging-related fibrosis by targeting the TGF-β signalling pathway [151-153] (Table 1). Saponins from *Panax japonicus* (SPJ) exert anti-inflammatory and antioxidative effects. Aging Sprague-Dawley rats were given SPJ by gavage beginning at 18 months of age. After 6 months, the treated group showed increased levels of MMP-2 and MMP-9, reduced levels of TIMP-1, TIMP-2, and TGF-β/Smad signalling, improved renal fibrosis and enhanced renal function. SPJ could be a potential drug for the treatment of aging-related renal fibrosis [151]. In addition, mangiferin is a well-known C-glucoside flavonoid found widely in herbs and fruits, including mango leaves *(Mangifera indica Linn*). Mangiferin has various beneficial properties, including antiapoptotic, antioxidant, and anti-inflammatory properties. Studies have shown that mangiferin ameliorates D-galactose-induced cardiac fibrosis in aging rats by inhibiting the TGF-β1/p38/MAPK signalling pathway, reducing cardiac oxidative stress, inflammation, and fibrosis [152]. Amylase Potato Protein Hydrolysate (APPH), a functional food associated with cardioprotection in high-fat diet model rats, can hydrolyse some proteins, such as soybeans, into antioxidant hydrolysates. Gelsolin (GSN) induces cardiac hypertrophy. After SD rats were fed a high-fat diet for 22 months, the expression of TGF-β and GSN in aging rats was measured; then the rats were treated with APPH, and APPH treatment significantly reduced aging- and high-fat diet-induced hypertrophy and fibrosis. Research indicates that APPH improves high-fat diet- and aging-induced cardiac hypertrophy and fibrosis induced by TGF-β-activated GSN [153].

Metformin is a biguanide, which is a drug class of herbal origin that has been widely used to treat diabetes since the 1950s [154]. Metformin reduces the loss of the epithelial marker E-cadherin in TGF-β-induced MCF-7 breast cancer cells and inhibits TGF-β-induced cell dispersion and the accumulation of the mesenchymal marker vimentin in Madin-Darby canine kidney cells. Studies have shown that metformin ameliorates aging-related fibrosis (e.g., chronic kidney disease, nonalcoholic steatohepatitis, cardiac failure, or sclerosis) and malignancy progression by impairing the ability of TGF-β signalling to adequately induce mesenchymal cell states in various pathological processes, reducing TGF-β-mediated inflammation and immune inflammation [155].

Date palm (*Phoenix dactilyfera L.*) seeds are rich in polyphenols and flavonoids. These agents have anti-inflammatory, immunostimulatory, antidiabetic, antibacterial, antiviral, and antioxidant pharmacological activities. Date palm seeds can inhibit inflammation by reducing the expression of IL-1β, TGF-β, cyclooxygenase-1, and -2. Date palm seeds can increase immunity, prevent chronic diseases, and improve aging-related fibrosis [156].

Xiaoyu Xiezhuo Drink (XXD) is derived from the “Buyang Huanwu Decoction” in the book “Yilin Gaicuo” and consists of Astragalus membranaceus (15-g), processed Radix cyathulae (6-g), Peach Kernel (6-g), Lumbricus (6-g), prepared Rhubarb (5-g), and Plantain (10-g). Studies have shown that XXD has nephroprotective effects on acute kidney injury in aged mice by inhibiting the TGF-β1/Smad3 signalling pathway. XXD significantly alleviates abnormal renal pathological changes, cellular senescence, inflammation, and oxidative damage and reduces the expression of inflammatory mediators. α-SMA, collagen-1, TGF-β1, Smad3 and p-Smad3 expression levels were reduced, and renal tissue MMP-9 and Smad7 levels were increased [157]. XXD can be used to improve aging-related renal fibrosis.

AANG is a natural combination of the Smad3 inhibitor naringenin and the Smad7 activator asiatic acid [158]. Smad3 is a key downstream mediator of fibrosis. Smad7 also inhibits NF-κB signalling by inducing IκBα and inhibits NF-κB-dependent renal inflammation, including diabetes [159]. AANG can ameliorate renal inflammation and aging-associated renal fibrosis.

## 7. Concluding remarks

The in-depth properties of TGF-β signalling have been extensively described. TGF-β signalling has long been recognized as a critical pathway in the development of fibrosis. As outlined in this review, an increasing number of studies have demonstrated that TGF-β signalling plays a crucial role in aging-related tissue fibrosis. Thus, inhibiting TGF-β signalling may be a viable therapeutic strategy in the treatment of aging-related fibrosis. In this review, the biological role of TGF-β in aging-related fibrosis was examined in depth. Overactivation of TGF-β signalling can lead to aging-related fibrosis in a variety of organs. TGF-β signalling can impact the development of tissue fibrosis due to aging. In this review, we described many pieces of evidence that make it clear that TGF-β signalling is the target of vascular fibrosis, IPF, cardiac fibrosis, liver fibrosis, and renal fibrosis. In light of the critical role of TGF-β in the development of aging-related fibrosis, the development of therapeutic agents to inhibit aberrant TGF-β signalling is essential for the effective treatment of patients with aging-related tissue fibrosis. Studies have highlighted the development of novel TGF-β inhibitors that could prevent and treat fibrosis in the future. In this review, we discussed various strategies to target TGF-β signal transduction in the treatment of aging-related fibrosis. Continuous suppression of TGF-β function may lead to unacceptable toxicity because TGF-β plays many roles. We should develop better strategies to overcome these adverse events. Local inhibition of TGF-β signalling or targeting downstream signalling molecules in the TGF-β signalling pathway may reduce adverse effects.
